# Potassium permanganate dye removal from synthetic wastewater using a novel, low-cost adsorbent, modified from the powder of *Foeniculum vulgare* seeds

**DOI:** 10.1038/s41598-022-08543-z

**Published:** 2022-03-16

**Authors:** Suhair. A. Bani-Atta

**Affiliations:** Department of Chemistry, Faculty of Science, University of Tabuk, Tabuk, 71491 Saudi Arabia

**Keywords:** Environmental chemistry, Environmental sciences, Chemistry, Materials science

## Abstract

In this study, Seeds powder of *Foeniculum vulgare* was used to prepare a novel adsorbent, the modification of the prepared adsorbent was done by each of ZnCl_2_, oxalic acid, and CuS, all samples have been characterized by different techniques and examined for Potassium permanganate (KMnO_4_) adsorption. Among the four modified and unmodified adsorbents, the sample modified by oxalic acid has the highest percentage removal for KMnO_4_ adsorption (%R = 89.36). The impact of KMnO_4_ concentration, adsorbent dose, contact temperature, contact time, and solution pH on the adsorption performance was also investigated. The experimental data of this adsorption was analyzed by different kinetic and isotherm models. As Constants of thermodynamic ΔG°, ΔH°, and ΔS° have been also evaluated. Surface area, pore volume, and pore size of the modified oxalic acid *F. vulgare* seeds powder adsorbent were determined as 0.6806 m^2^ g^−1^, 0.00215 cm^3^ g^−1^, and 522.063 Å, as pH_ZPC_ also was stated to be 7.2. The R^2^ values obtained from applying different isotherm and kinetic models (0.999 and 0.996) showed that the adsorption performance of KMnO_4_ follows the Langmuir and Pseudo 2nd order models. Furthermore, high adsorption capacities of 1111.11, 1250.00, and 1428.57 mg g^−1^ were achieved at three temperatures that were used in this study. Constants of thermodynamic ΔG°, ΔH°, and ΔS° values indicate chemical and spontaneous adsorption at the adsorbent surface. Therefore, the modified adsorbent can be used to remove KMnO_4_ dye from pollutant water samples.

## Introduction

Potassium permanganate (KMnO_4_) is a highly strong oxidizing agent that is commonly used for water purification from numerous pollutants, mainly for the destruction of compounds that cause undesirable taste, odor, and color for the treated water^1^. Remarkably, permanganate is still one of the most oxidizing chemicals ever applied to remove each iron, manganese, and arsenic from water^2,3^, In addition to its great ability to oxidize cyanide, phenols, and organic compounds^4–8^.

Recently, many studies reported that excessive exposure to KMnO_4_ may cause acute problems of the nervous system, irritation of the skin and eye, Furthermore, it was stated that manganese has significant toxicity towards the liver and kidneys^9,10^. Therefore many techniques and methods have been applied for KMnO_4_ removal from the contaminated water. For instance, fluidized-bed crystallization method was used^11^, concerning the ease of preparation and use, in addition to the high capacity to get rid of permanganate from wastewater adsorption is one of the most extensively utilized processes with different adsorbents^1^, activated carbon is a common adsorbent that used for adsorption of KMnO_4_ from polluted water due to its high adsorption efficiency^12,13^.

To remove KMnO_4_ molecules from polluted aqueous solutions by adsorption a lot of activated carbon adsorbents were prepared using shells of coconut^12^, corn cob, and animal bone were also applied^14^, sulfuric acid modification of activated carbon, and activated charcoal have been used too^1,15^. More recently, Nanoparticles of metallic oxides have been used to remediate effluent from various dyes^16–22^, Copper sulfide nanoparticles were used as dynamic adsorbents to treat the synthetic wastewater from potassium permanganate ions ^23^.

Despite the great performance and significant efficiency of activated carbon and metallic oxides Nanoparticles, its requirements and conditions of preparation are rather difficult and expensive. Thus, prompted the researchers to use low-cost materials within their areas and applied them as adsorbents for permanganate ions adsorption. For instance, sage^24^ Neem^25^, Nitraria retusa^26^, and Ocimum basilicum^27^ were used as low-cost sorbents to remove Permanganate anions from synthetic samples.

*Foeniculum vulgare* plant is well-known by fennel in many countries as shamr in Saudi Arabia, mainly used as food and tea flavored and considered as a flavored spice, its seeds were used as antitumor^28^, antimicrobial^29^, and antioxidant^30^. Experiments on animals and clinical trials recommend that chronic use of *F. vulgare* plant is not harmful and no toxicity marks were detected ^31^.

Gold nanoparticles based on seeds extract of fennel *F. vulgare* plant was synthesized and its catalytic activity against rhodamine B and methylene blue days were examined^32^, V_2_O_5_–Fe_2_O_3_ nanocomposites from stem powder of *F. vulgare* have been also produced and the catalytic performance of nanocomposites particles was assessed for reduction of 4-nitrophenol^33^.

Up to now, no adsorbent based on *F. vulgare* seeds was prepared in any form and applied to eliminate the hazardous dyes from water even permanganate ions, despite the excellent medical properties of this herb, in addition to its widespread over the world *F. vulgare* seeds are considered a low-cost material. Therefore, this research mainly aimed to prepare a new adsorbent from seeds of *F. vulgare* and to investigate the adsorbent performance toward eliminating KMnO_4_ from polluted water. Thermodynamics, Kinetics, and isotherms parameters will also be studied. The performance of this adsorption will be also studied through conditions and impacts that could affect on KMnO_4_ removal experiment, as the adsorption capacity of modified adsorbent for removal of KMnO_4_ will be critically addressed.

To achieve all the desired goals of this work and get the best results, unmodified samples from *F. vulgare* seeds have been synthesized and the modification has been also carried out by zinc chloride, copper sulfide, and oxalic acid; both types of samples have been characterized and examined as adsorbents for KMnO_4_ removal from water, to choose the best adsorbent, the adsorption performances have been compared, then all the KMnO_4_ adsorption experimental factors and conditions of selected adsorbent were tested.

## Materials and methods

### Materials

*Foeniculum vulgare* seeds were obtained from a local market in Tabuk City, KSA. All chemicals that were used in this work were obtained from Sigma-Aldrich with a purity of (37%) for hydrochloric acid, ≥ 97% for sodium hydroxide ≥ 97% for zinc chloride, ≥ 99.99% for oxalic acid, ≥ 99:99% for copper sulfide, and ≥ 99.00% for sodium carbonate.

### Preparation and modification of adsorbents

The *F. vulgare* seeds were washed with distilled water several times and then dried overnight, after that, the *F. vulgare* seeds powder (FVESP) was obtained by an electric grinder. A sample of 100 g was refluxed for 180 min with 1 L of oxalic acid (20% w/w), afterwards, the mixture was allowed to cool at room temperature. The sold part was separated by filtration, to get rid of any excess amount of oxalic acid; the solid was heated for 90 min with 250 mL of 2 M hydrochloric acid. Then, the filtration of the new mixture was done many times and rinsed with distilled water to have a clean solid, to get rid of any water present in the sample; the solid was left in the oven for 30 h at 130 °C. Finally, to ensure the homogeneity of the sample, the dry solid was grinded and sieved, and the resulted adsorbent of oxalic acid *F. vulgare* seeds powder labeled as (Ox-FVESP).

The same procedure was repeated with mixtures containing 100 g of FVESP with 1 L of 20% w/w acidic solution of zinc chloride, and 100 g FVESP with a mixture of 20% w/w acidic solution of zinc chloride and 50 g of copper sulfide. The resulted adsorbents of zinc chloride *F. vulgare* seeds powder labeled as (Zn-FVESP), and zinc chloride/copper sulfide *F. vulgare* seeds powder (Zn/Cu-FVESP).

### Characterization of FVESP adsorbents

To recognize the surface morphology of the modified and unmodified FVESP adsorbents SEM instrument was used at a 10 kV accelerating voltage. And to determine the surface adsorbents' functional groups, the FT-IR instrument (Nicolet iS5 of Thermo Scientific FT-IR, USA) was carried out. The surface area and porosity of each adsorbent were estimated using BET (NOVA-2200 Ver. 6.11) technology for 22 h and 77.35 K. In addition, 40 mL of 0.05 M Na_2_CO_3_ solutions varying with 2, 4, 6, 8, and 10 initial values of pH_i_ have been mixed in a 150 mL plastic container with 0.2 g of the idealistic adsorbent. After shaking all containers for 26 h 175 rpm and 27 °C conditions in a shaker incubator, filtration of each was done, then using a pH meter, the final pH (pH_f_) of each solution was determined. Finally, to determine the pH_ZPC_ value of this adsorbent, the values of (pH_i_ − pH_f_) have been calculated and graphed against the pH_i_ values.

### Adsorption experiments

#### The idealistic adsorbent identification

To determine the superlative as well as the most efficient adsorbent developed in the current study for KMnO_4_ removal from synthetic aqueous samples, 20 mL of 100 mg L^−1^ KMnO_4_ solution concentration was combined with 0.03 g of FVESP in a 30 mL amber bottle. A shaker incubator was used for 30 h to stir the sealed amber bottle at 27 °C and 180 rpm. After that, the mixture was filtered; The Jenway UV-6800 UV–Vis spectrophotometer was used at 525 nm to measure the balanced concentration of KMnO_4_ in the filtrate. The same procedure was repeated with Ox-FVESP, Zn-FVESP, and Zn/Cu-FVESP adsorbents for the KMnO_4_ adsorption. Equations (1) and (2) were used to calculate the KMnO_4_ percentage removal percent %R and the quantities of KMnO_4_ adsorbed at equilibrium Q_e_ mg g^−1^ by both modified and unmodified adsorbents.

1 $$\%R=\frac{C-C}{C} 100\%$$

2 $$ \begin{gathered} Q_{e} = \frac{\,V}{m}\left( {C_{ \circ } - C_{e} } \right)\, \hfill \\ \, \hfill \\ \end{gathered} $$

where C_∘_ is the KMnO_4_ initial concentration and C_e_ is the KMnO_4_ final concentration, m, and V are the mass of adsorbent (g), and KMnO_4_ solution volume (L), respectively.

### Experimental conditions impact

Batch experiments have been conducted to observe and identify the most significant factors that affect KMnO_4_ adsorption experiments by ideal adsorbent Ox-FVESP, such as concentration of KMnO_4_ (10–1400 mg L^−1^), contact time (0–320 min), the dosage of Ox-FVESP adsorbent (0.005–0.035 g), the adsorption temperature (27–57 °C), and the pH (1.5–11.5). All of the Batch experiments have been done in 30 mL amber bottles by adding 20 mL of KMnO_4_ solution to enough amounts from Ox-FVESP. A shaker incubator at 180 rpm was used to shake all sealed amber bottles for a required time, followed by filtration of each mixture, and the remaining concentrations of KMnO_4_ were measured as mentioned previously.

To compute the adsorbed amount of KMnO_4_ at equilibrium (Q_e_, mg g^−1^) by the Ox-FVESP adsorbent and time t (Q_t_, mg g^−1^) Eqs. (2) and (3) were applied.

3 $$ \begin{gathered} Q_{t} = \frac{\,V}{m}\left( {C_{ \circ } - C_{t} } \right)\, \hfill \\ \, \hfill \\ \end{gathered} $$

where C_t_ (mg L^−1^) is the KMnO_4_ concentration of at contact time.

### Temperature impact and isotherm studies

The outcomes obtained from the Batch experiments for 10–1400 mg L^−1^ KMnO_4_ solutions by 0.02 g by Ox-FVESP adsorbent at 26 h contact time and three different temperatures (27, 42, and 57 °C) and 190 rpm have been analyzed according to the three isotherm models, Langmuir, Freundlich, and Temkin linear forms, Eqs. (4–6) respectively. The parameter of equilibrium R_L_ value of the Langmuir isotherm model was also evaluated according to Eq. (7).

4 $$ \frac{{C_{e} }}{{q_{e} }} = \frac{1}{{q_{\max } K_{L} }}\,\, + \frac{{C_{e} }}{{q_{\max } }}\,\,\,\,\,\, $$

5 $$ \ln q_{e} = \ln K_{F} + \frac{1}{n}\ln C_{e} $$

6 $$ q_{e} = B_{1} \ln K_{T} + B_{1} \ln C_{e} $$

7 $$ \begin{gathered} R_{L} \, = \,\frac{1}{{1 + K_{L} \,C_{0} }}\,\,\,\,\, \hfill \\ \hfill \\ \end{gathered} $$

where C_o_ is the maximum initial concentration of KMnO_4_ and K_L_ is the constant of Langmuir, K_F_ is the constant of Freundlich, and K_T_ is the constant Temkin. q_max_ (mg g^−1^) is the maximum capacity of adsorption. B1 and n are constants of the adsorption heat and the intensity of adsorption, respectively.

### Contact time impact and Kinetic studies

The experimental data obtained from the adsorption Batch experiments, adsorption of KMnO_4_ by Ox-FVESP with a concentration of 50, 100, and 200 mg L^−1^ at several times from 0 to 320 min and 27 °C and 190 rpm have been analyzed by three of different kinetic models. Equations (8)–(10), Pseudo 1st order, Pseudo 2nd order, and Intraparticle diffusion, correspondingly. Then, the achieved results have been used to study each of the conducted time impact, rate, and mechanism of KMnO_4_ adsorption by Ox-FVESP adsorbent.

8 $$ \log (Q_{e} - Q_{t} ) = \log Q_{e} - K_{1} \frac{t}{2.303}\,\,\,\,\, $$

9 $$ \frac{t}{{Q_{t} }} = \frac{1}{{K_{2} Q^{2}_{e} }} + \frac{t}{{Q_{e} }} $$

10 $$ \mathop Q\nolimits_{t} = \,K_{dif} \sqrt t \,\, + C\,\, $$

Q_t_ (mg g^−1^): the amount of adsorbed KMnO_4_ at time t, Q_e_: the amount of adsorbed KMnO_4_ at equilibrium, K_1_ (1 min^−1^): rate constants of Pseudo 1st order, K_2_: (g mg^−1^ min^−1^) rate constants of the 2nd order. K_dif_ (mg g^−1^ min^−1^)^1/2^ and C are rate constants of intraparticle diffusion.

### Thermodynamic experiment

Constants of thermodynamic ΔG°, ΔH°, and ΔS° have been also evaluated from the outcomes of experimental Conditions impact part for adsorption of 500, 700, 1000, and 1200 mg L^−1^ KMnO_4_ solutions according to Eqs. (11) and (12).

11 $$ Ln\left( {\frac{{Q_{e} }}{Ce}} \right) = - \frac{{\Delta \mathop {\text{H}}\nolimits^{^\circ } }}{RT} + \frac{{\Delta \mathop S\nolimits^{^\circ } }}{R} $$

12 $$ \mathop {\Delta G}\nolimits^{ \circ } = \mathop {\Delta {\rm H}}\nolimits^{ \circ } - \mathop {T\Delta S}\nolimits^{ \circ } \, $$

where ΔS°, ΔG°, and ΔH° are the change in standard entropy, change in standard free energy, and is the change in standard enthalpy, T and R are the adsorption temperature of (K) and universal gases constant (8.314 J K^−1^ mol), respectively.

## Results and discussion

### FVESP characterization

**Figure 1:**
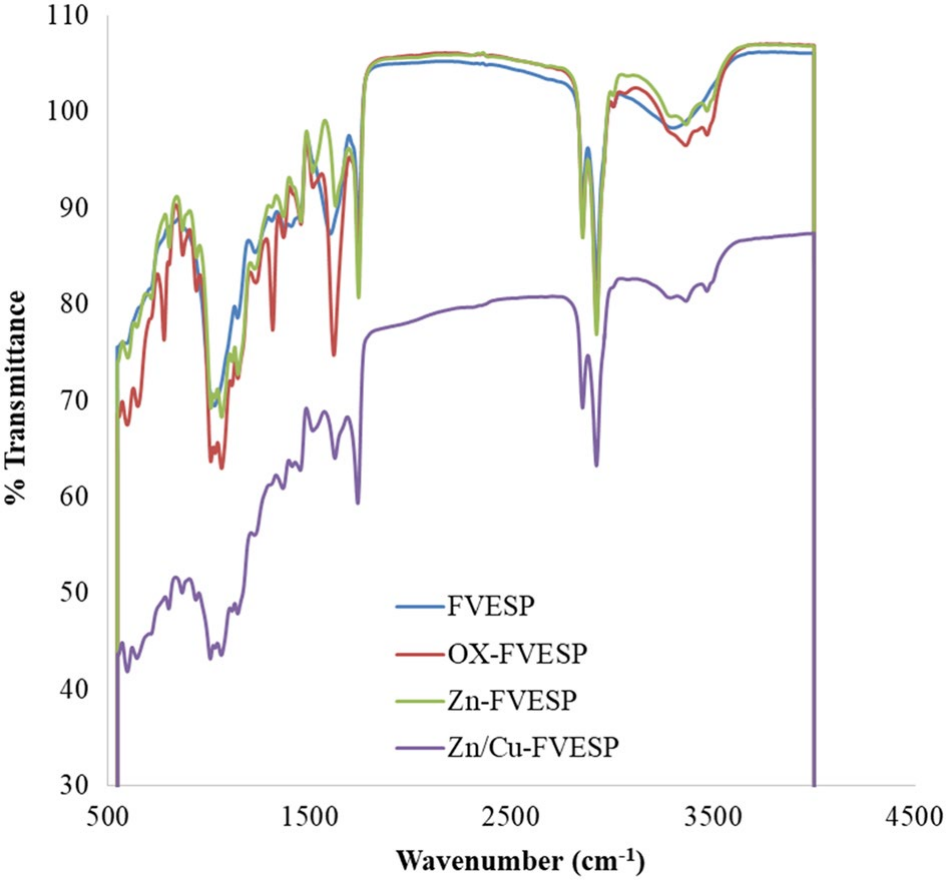
FT-IR for the FVESP and Ox-FVESP and Zn-FVESP and Zn/Cu-FVESP.

The FT-IR spectra of four samples of modified and unmodified FVESP are revealed in Fig. 1. It can be observed from the figure that the unmodified FVESP sample has six peaks at 1060 cm^−1^ for C–O stretching, 1118 cm^−1^ for C–O stretching of a secondary alcohol, 1025 cm^−1^ for C–F stretch Aliphatic fluoro compounds, 1590 cm^−1^ for C=C stretching, 2870 cm^−1^ and 2940 cm^−1^ for stretching the C–H alkane, and 3360 cm^−1^ for hydrogen bond stretching of the O–H. Figure 1 illustrates also that the modified Zn-FVESP, and Zn/Cu-FVESP adsorbents showed the same peaks with a slight shift, while in the case of Ox-FVESP sample, many peaks developed (Fig. 1), and these bands are 1190 cm^−1^, 1320 cm^−1^, and 1620 cm^−1^, The appearance of these bands support the success of the chemical modification process that was carried out for the adsorbent and also confirms the variety of functional groups on the surface of Ox-FVESP, which will have an effective role in permanganate adsorption from the water later.

**Figure 2:**
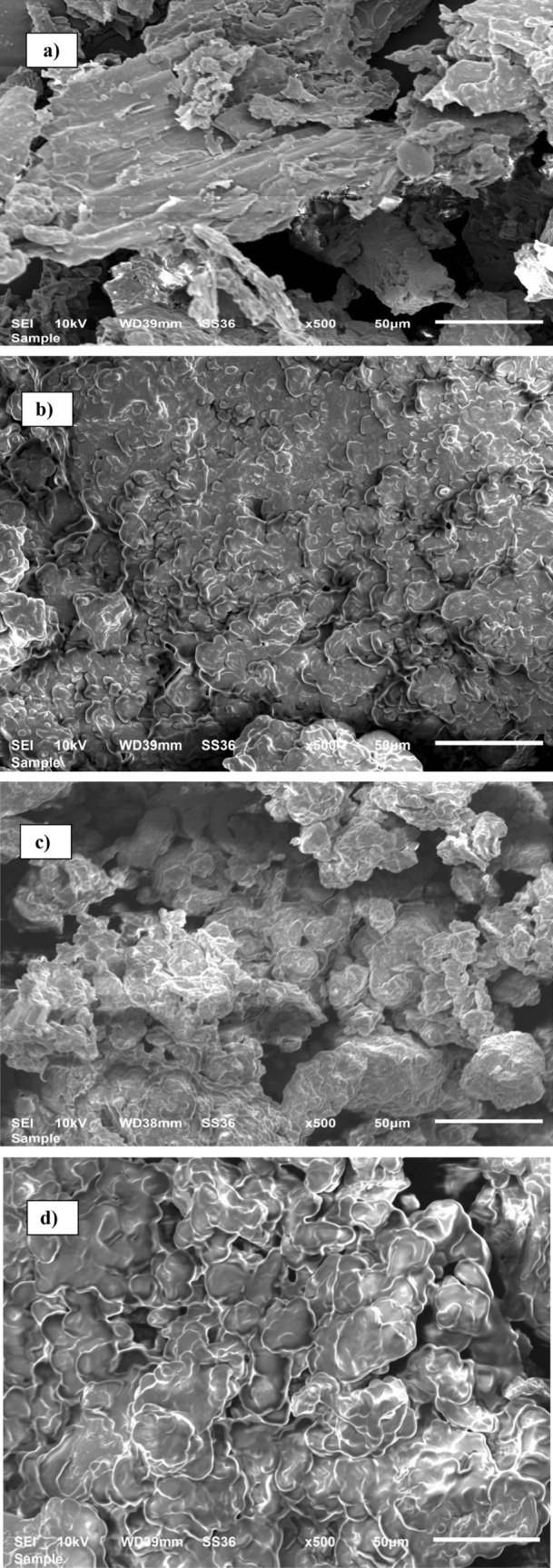
SEM images of (**a**) FVESP and (**b**) Zn-FVESP and (**c**) Ox-FVESP and (**d**) Zn/Cu-FVESP.

The spectrum of FVESP, Zn-FVESP, Ox-FVESP, and Zn/Cu-FVESP SEM images are demonstrated in Fig. 2a–d, respectively. When comparing the SEM images of modified samples (b), (c), and (d) to the unmodified adsorbent (a), it can be seen that the surface of the FVESP adsorbent has been significantly transformed by modification procedure, as most of the modified adsorbents pleats have been distorted and their structures became scattered. Furthermore, several heterogeneous holes and pores have appeared on the modified adsorbents surfaces, which improve the adsorption performance. It is also recognized from Fig. 2c that the density of micropores of the modified adsorbent is more than the rest of the other samples.

**Figure 3:**
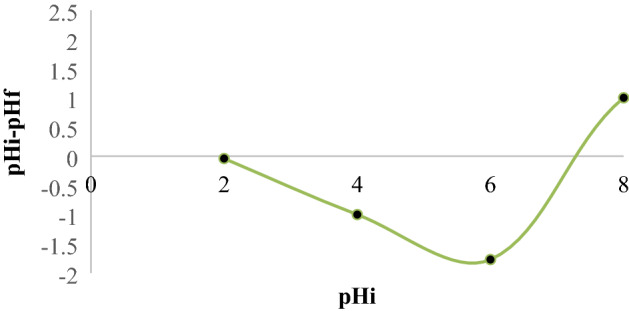
pH_ZPC_ of the Ox-FVESP adsorbent.

The relationship between pHi and pHi–pHf is depicted in Fig. 3, which shows that pH_ZPC_ (the solution pH when the surface of sorbent has a zero net charge) is 7.2. Meanwhile, the surface charge of the adsorbent will be positive and negative at solution pH levels lower and higher than 7.2, Al-Aoh^25^ has previously found similar findings.

BET surface analyzer results for the modified and unmodified FVESP samples are listed in Table 1, Surface Area (m^2^ g^−1^), Volume of Pore (cm^3^ g^−1^), and Size of Pore (Å). The table shows that the Ox-FVESP sample achieved the highest surface area (0.6806 m^2^ g^−1^) and size of the pore (522.063 Å) compared to the rest of the other samples, The highest values of the surface area and size of pore will positively affect the process of permanganate adsorption on the modified FVESP surface by oxalic acid and prove that the modification process has an important and obvious role.

**Table 1:** BET surface analyzer of FVESP, Ox-FVESP, Zn-FVESP, and Zn/Cu-FVESP.

Sample	Surface area (m^2^ g^−1^)	Volume of pore (cm^3^ g^−1^)	Size of pore (Å)
FVESP	0.0249	0.00153	53.001
Ox-FVESP	0.6806	0.00215	522.063
Zn-FVESP	0.3087	0.00214	91.717
Zn/Cu-FVESP	0.3979	0.00061	91.037

### The idealistic adsorbent identification

**Figure 4:**
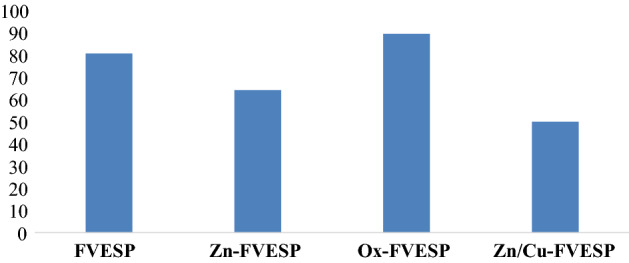
The percentage removal for KMnO_4_ adsorption by four different samples.

Figure 4 illustrates the percentage removal for KMnO_4_ adsorption by four different samples that were synthesized and modified in this work, and it was as the following 80.52 for FVESP, 64.03 for Zn-FVESP, 89.36 for Ox-FVESP, and 49.08 for Zn/Cu-FVESP. The percentage removal values show that the Ox-FVESP adsorbent has the greatest percentage among other samples, so, these findings support the Ox-FVESP adsorbent is the best sample for KMnO_4_ adsorption. Also, these results were fully consistent with the SEM and BET surface outcomes. As a result, only Ox-FVESP adsorbent was used in the rest of this study.

### Experimental conditions impact

#### Influence of pH solution

**Figure 5:**
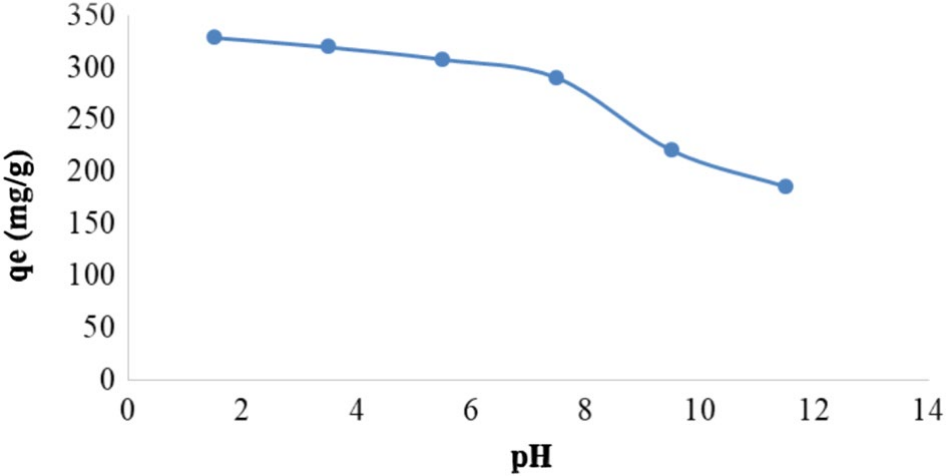
The pH solution impact on the of KMnO_4_ adsorption by Ox-FVESP adsorbent.

The adsorption performance is greatly influenced by the pH of the adsorbate solution, degree of ionization, and charge of adsorbent of the dye molecules also impacted by pH. As a result, the impact of this issue was addressed in this study (Fig. 5). It is clear from the figure that the *qe* (mg g^−1^) value was greatly affected by the pH values, as it was high when pH values were raised from 1.5 to 7.2, and this is due to the high attraction between the positive charges of the Ox-FVESP surface and the MnO_4_^−^ anions. In contrast, increasing the pH value over 7.2 has a negative effect on *qe* (mg g^−1^) because of the significant repulsion between the negative MnO_4_^−^ ions and the negative charges of this adsorbent surface. Like results have been found for the KMnO_4_ elimination by chemically modified sage leaves powder^24^.

### ***Impact of Ox***-***FVESP doses***

**Figure 6:**
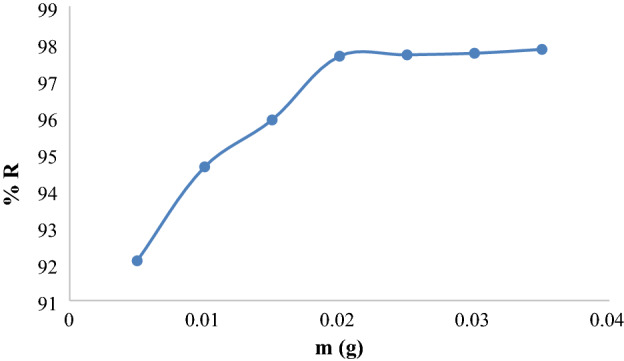
Effect of dose on adsorption performance of Ox-FVESP for KMnO_4._

To specify the ideal mass of Ox-FVESP that will be required for the KMnO_4_ adsorption the percent removal of KMnO_4_ was plotted against Ox-FVESP doses (Fig. 6). The values of percent removal of KMnO_4_% R are improved by increasing the mass of Ox-FVESP from 0.005 to 0.020 g. This rise was caused by the improvement of the active sites on the Ox-FVESP surface, which is related to the adsorbent quantity^34^. Figure 6 shows also the percent R value does not change significantly when the mass of the adsorbent is increased from 0.020 to 0.035 g and it is assumed that the amount of dye adsorption was significantly affected by the concentration of unfilled dynamic reactive sites due to the bonding ability of the adsorption surface function^35,36^. In this study, 0.020 g of Ox-FVESP was chosen as the optimal dose. The adsorption of CR dye by Zn/Cu-TPLLP adsorbent^23^ and KMnO_4_ on the CuS surface showed a similar Patten^37^.

### Temperature impact and isotherm studies

**Figure 7:**
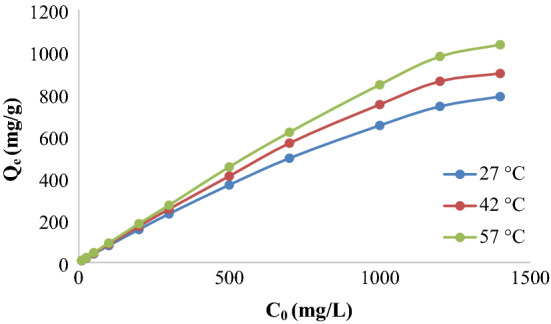
Impacts of initial concentration and temperature on the KMnO_4_ adsorption by Ox-FVESP.

The impact of initial solution concentration and temperature on the adsorption capacity of this work is demonstrated in Fig. 7. Figure 7 shows the relationship between the adsorption amount Q_e_ (mg/g) and the concentration of KMnO_4_ (10–1400 mg L^−1^) at 27, 42, and 57 °C temperatures. It can be observed from the figure that raising the temperature of the solution has a positive impact on the adsorption capacity of KMnO_4_ by Ox-FVESP. And this refers to the decreasing of KMnO_4_ viscosity with solution temperature increasing, also, the kinetic energy of the permanganate particles increases with rising the temperature; the same kinetic energy performance for permanganate ions was recorded by neem leaves powder adsorbent^25^. It is also noted from the same figure that the adsorption of permanganate is improved by raising the concentration of KMnO_4_ from 10 to 1400 (mg L^−1^) at the same temperature. And this could be supported by the finding that raising the adsorbate concentration will develop the dynamic force^38^, which lowers the resistance of KMnO_4_ particles mass movement between the Ox-FVESP surface and adsorbate solution. It is also clear that the adsorbent will be effective even at KMnO_4_ concentrations higher than 1400 mg L^−1^, and this is refer to the unfilled adsorption sites on the adsorbent surface.

**Figure 8:**
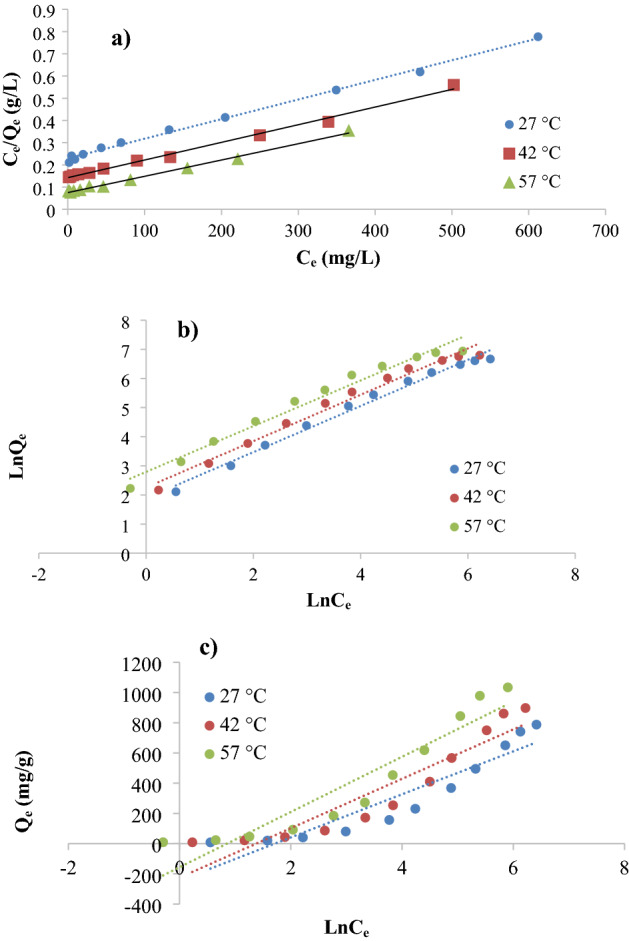
(**a**) Langmuir and (**b**) Freundlich and (**c**) Temkin isotherm models for adsorption of KMnO_4_ by Ox-FVESP.

Moreover, The outcomes obtained from Batch experiments were analyzed according to the isotherm model of Langmuir (Ce against Ce/qe), isotherm model of Freundlich (ln Ce against ln qe), and isotherm model of Temkin (ln Ce against qe) Fig. 8a–c, the slopes and intercepts of these plots were used to achieve the isotherm parameters and presented in Table 2. Where the experimental results are well fitted by applying the Langmuir isotherm model and the R^2^ values were the highest compared with Freundlich and Temkin models Table 2, which approves that the Langmuir model is the best fit for this adsorption. These findings also show that the adsorption of KMnO_4_ is monolayer adsorption and that the Ox-FVESP adsorption sites are homogeneous. Same outputs for KMnO_4_ adsorption as recorded by a chemical modified powder from leaves of neem^25^. Moreover, the Favorable adsorption was confirmed by values of R_L_ which ranged between 0 and 1^14^.

**Table 2:** Isotherm constants of Langmuir and Freundlich and Temkin models for KMnO_4_ adsorption by Ox-FVESP.

Temperature	Isotherm parameters										
	Langmuir				Freundlich				Temkin		
	q_max_ (mg g^−1^)	K_L_ (L mg^−1^)	R_L_	R^2^	K_F_ (mg g^−1^) (L mg^−1^)^1/n^	1/n	n	R^2^	K_T_ (L mg^−1^)	B_1_	R^2^
27 °C	1111.11	0.00392	0.15408	0.996	6.2557	0.792	1.2625	0.988	0.18027	142.58	0.871
42 °C	1250.00	0.00561	0.11294	0.994	9.5735	0.796	1.2560	0.982	0.25209	163.59	0.878
57 °C	1428.57	0.00928	0.07144	0.994	13.6154	0.785	1.2740	0.975	0.42520	182.81	0.886

Furthermore, high adsorption capacities of 1111.11, 1250.00, and 1428.57 mg g^−1^ (Table 2) were achieved, at three temperatures that were used in this study. This demonstrates that Ox-FVESP, as a low-cost and very effective adsorbent, will be of particular importance in the purification of wastewaters from the KMnO_4_.

### Contact time impact and Kinetic studies

**Figure 9:**
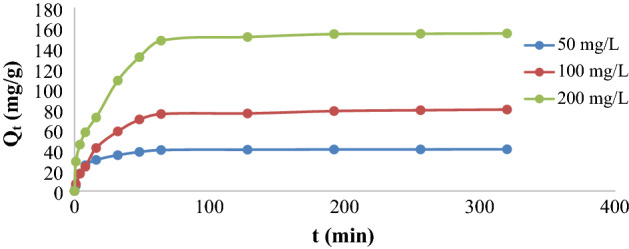
Impact of adsorption time on KMnO_4_ adsorption by Ox-FVESP.

To investigate the contact time impact on the KMnO_4_ adsorption experiment, the contact time (t) has been graphed against Q_t_ (mg g^−1^) (adsorption quantity at such time t) for the adsorption of (50, 100, and 200 mg L^−1^) KMnO_4_ concentrations by the ideal dose of chemically modified FVESP adsorbent selected for this work (Fig. 9). Figure 9 shows that there are three adsorption regions, the higher adsorption was detected at region I (0–16 min) where the adsorption amount (Q_t_) rapidly augmented, while the increase in region II (16–64 min) was regularly and after 64 min till the end of the experiment time, it was practically consistent (region III). Initially, the removal rate was high due to the availability of the abundant of functional groups^39^. Similarly, the sharp increase in the rate of removal at the beginning of the adsorption process showed the strong attraction forces between OX-FVESP sites and KMnO_4_. The same results for KMnO_4_ adsorption were obtained by a powder of sage leaves modified by zinc chloride^24^ and for the adsorption of Cu(II) ions on the nanomaterials surface adsorbent^40^. It is also noted from the same figure that the equilibrium time occurred at the 45th minute of the experiment time.

**Figure 10:**
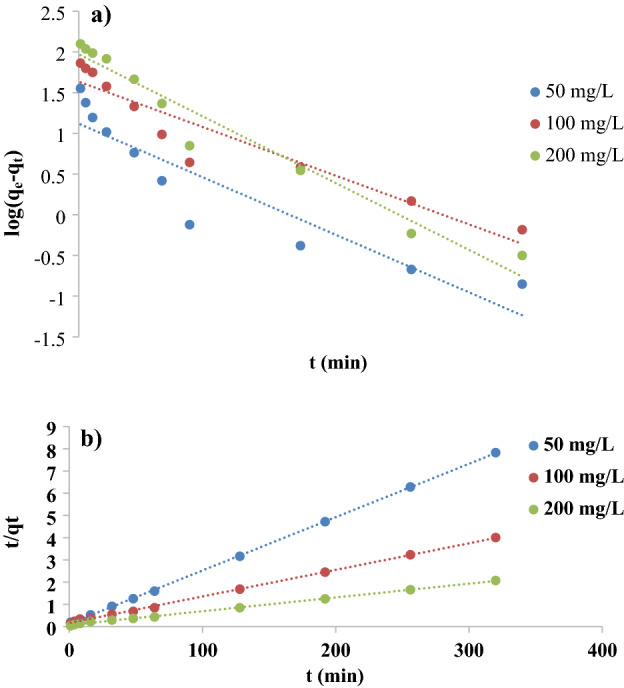
(**a**) Kinetic model of the 1st order and (**b**) Kinetic model of the 2nd order for KMnO_4_ adsorption by Ox-FVESP.

**Figure 11:**
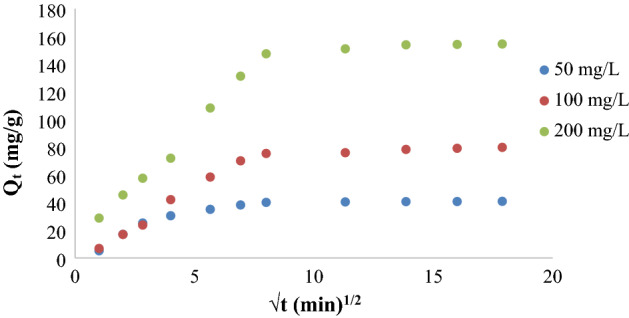
Model of intra-particle diffusion for KMnO_4_ adsorption by Ox-FVESP.

Furthermore, the experimental outcomes of this adsorption have been studied according to Pseudo-first order, pseudo-second order, and Intraparticle diffusion kinetics models Figs. 10a,b, and 11. The slopes and intercepts of these plots were used to calculate the kinetic parameters and summarized in Tables 3 and 4, the linear relationships observed by applying the pseudo-second order model in Fig. 10b, where the highest R^2^ values occurred, and the good agreement between the experimental Q_e_ values (Table 3) and computed values of Q_e_, which approve that the adsorption of this work followed the second-order kinetic model. And implying that the biosorption of KMnO_4_ from the aqueous media is governed by a chemical kinetic mechanism involving electron exchange or sharing between the anionic part of the dye (MnO_4_^−^) and the functional groups on the OX-FVESP adsorbent surface. Similar findings were stated for KMnO_4_ adsorption by activated carbon^12^, nanoparticles prepared from copper sulfide^23^, and powder sage leaves modified by zinc chloride^24^. The dye adsorption process by modified activated carbon adsorbents followed also the pseudo-second order^41,42^.

**Table 3:** Parameters of the 1st and 2nd-order kinetic models for adsorption of KMnO_4_ by Ox-FVESP.

C_0_ (mg L^−1^)	Q_e,exp_ (mg g^−1^)	Kinetic model						
		1st order			2nd order			
		Q_e1,cal_ (mg g^−1^)	K_1_ (h^−1^)	R^2^	Q_e2,cal_ (mg g^−1^)	K_2_ (g mg^−1^ h^−1^)	R^2^	Rate
50	40.89	13.14	0.0212	0.85	41.67	0.0046	0.999	0.19308
100	79.87	43.14	0.0180	0.898	83.33	0.0009	0.999	0.07481
200	154.57	93.86	0.0246	0.958	161.29	0.0005	0.998	0.08832

**Table 4:** Parameters of the intra-particle-diffusion kinetic model for KMnO_4_ adsorption by Ox-FVESP.

*C*_*o*_ (mg L^−1^)	First region			Second region		
	*K*_*dif*_ (mg h^−1/2^ g)	*C*	*R*^*2*^	*K*_*dif*_ (mg h^−1/2^ g)	*C*	*R*^*2*^
50	5.187	5.839	0.889	0.0743	39.59	0.973
100	10.990	9.413	0.993	0.4868	71.27	0.945
200	17.271	− 4.389	0.982	0.7332	142.44	0.900

Intra-particle diffusion plots for KMnO_4_ adsorption by Ox-FVESP (Fig. 10) and R^2^ values, Table 4 display that the relationship between contact time (t) and adsorption amount (Q_t_) could not be linear at all, but two different areas are observed. Furthermore, all the plots do not cross the original and this approves that the adsorption of MnO_4_^−^ ions is not affected by the Intra-particle diffusion step; migration of MnO_4_^−^ ions via the Ox-FVESP pores will be very simple. This agrees with the SEM results, as it was clear that the Ox-FVESP surface has a lot of asymmetrical pores.

### Thermodynamic experiment

Equation (11) was applied to evaluate parameters of the thermodynamic ΔH°, and ΔS° at three different temperatures for solution Initial concentrations 500, 700, 1000, and 1200 mg L^−1^. Then, the values of ΔG° were computed according to Eq. (12) based on the previously calculated values of ΔS° and ΔH° and illustrated in Table 5. The lowering in the randomness and the endothermic process of permanganate adsorption by Ox-FVESP adsorbent was confirmed by the positive values of each ΔS° and ΔH° (Table 5)^26^. Moreover, the ΔH° values are higher than 20.9 kJ mol^−1^, ranging from 27.541 to 34.371 kJ mol^−1^, which indicates the molecules of adsorbate were chemically adsorbed at the adsorbent surface sites, these results are in agreement with the previous kinetic outputs. Negative values of ΔG° suggest spontaneous adsorption in the range of temperature that is used in this study, similar findings were stated for KMnO_4_ adsorption by modified Powder of Ocimum basilicum^26^ and other adsorbents developed from very low-cost adsorbents^23,24^, the adsorption process by cationic polymeric adsorbent also achieved similar results^43^.

**Table 5:** Thermodynamic constants for KMnO_4_ adsorption by Ox-FVESP.

Initial concentration (mg L^−1^)	∆H^o^ (kJ mol^−1^)	∆S^o^ (kJ mol^−1^)	∆G^o^ (KJ mol^−1^)			R^2^
			300 K	315 K	330 K	
500	34.371	0.1227	− 2.4260	− 4.2659	− 6.1057	0.977
700	31.346	0.1117	− 2.1758	− 3.8519	− 5.5280	0.999
1000	29.246	0.1024	− 1.4849	− 3.0214	− 4.5580	0.992
1200	27.541	0.0956	− 1.1374	− 2.5713	− 4.0051	0.992

### Comparative study with other adsorbents

Table 6 summarized the adsorption capacities of KMnO_4_ removal by Ox-FVESP at three temperatures and the capacities of other synthesized low-cost adsorbents. As presented in Table 6, Ox-FVESP adsorbent has a higher adsorption capacity than the conventional low-cost adsorbents that were previously employed to remove KMnO_4_ from aqueous samples. As a result, the cost-effectiveness of Ox-FVESP, easy availability, and its high performance in adsorption of permanganate from polluted water give this adsorbent a strong opportunity over other adsorbents.

**Table 6:** Adsorption capacities for KMnO_4_ removal by several adsorbents.

Adsorbant	Temperature (°C)	Q (mg g^−1^)	Reference
Ox-FVESP	27	1111.11	This study
	42	1250.00	
	57	1428.57	
Granular activated charcoal		57.47	^1^
Animal bone-derived activated carbon		28.04	^14^
Corncob derived activated carbon		26.00	^14^
Coconut shells derived activated carbon		23.25	^12^
Modified activated carbonaceous materials		100.00	^15^
Zinc chloride *Ocimum basilicum* leaves powder	25	588.235	^37^
	35	625.000	
	45	666.667	
	55	714.286	

## Conclusions

Modification of the *F. vulgare* Seeds (FVES) powder was done by each of ZnCl_2_, oxalic acid, and CuS, all samples have been characterized by different techniques and examined for permanganate (KMnO_4_) adsorption. Among the four modified and unmodified samples, the sample modified by oxalic acid (Ox-FVESP) has the highest percentage removal for KMnO_4_ adsorption (%R = 89.36), and was nominated as a new adsorbent for KMnO_4_ adsorption from the synthesized solutions. The surface area, volume, and size of the pore of the Ox-FVESP adsorbent were determined as 0.6806 m^2^ g^−1^, 0.00215 cm^3^ g^−1^, and 522.063 Å, respectively, as pH_ZPC_ also was stated to be 7.2. The influence of KMnO_4_ concentration, Ox-FVESP dose, pH of the solution, adsorption temperature, and adsorption time on the KMnO_4_ adsorption was inspected, it can be noted from the experimental outcomes the adsorption performance of KMnO_4_ was positively affected by the rising concentration of KMnO_4_ from 10 to 1400 mg L^−1^, Ox-FVESP dose from 0.005 to 0.020 g, contact temperature from 27 to 57 °C, and adsorption time from 0 to 64 min. While the increase of solution pH from 1.5 to 11.5 has a negative effect on the adsorption process. The calculated R^2^ values of different isotherm and kinetic models (0.999 and 0.996) revealed the adsorption performance of KMnO_4_ following the Langmuir and Pseudo 2nd order models. Constants of thermodynamic ΔG°, ΔH°, and ΔS° values indicate chemical and spontaneous adsorption at the adsorbent surface. Additionally, high adsorption capacities were accomplished at three temperatures that were used in this work 1111.11, 1250.00, and 1428.57 mg g^−1^. Proposing that the Ox-FVESP adsorbent prepared from very low-cost material was important to explore the use in the water purification from dye at optimum conditions.

## Data Availability

The datasets used and/or analyzed during the current study are available from the corresponding author on reasonable request. And I state that the experimental research on plant seeds used in this study complied with the relevant institutional, national, and international guidelines and legislation.
